# Leveling the playing field: Grounding learning with embedded simulations in geoscience

**DOI:** 10.1186/s41235-016-0026-3

**Published:** 2016-12-07

**Authors:** Allison J. Jaeger, Jennifer Wiley, Thomas Moher

**Affiliations:** 1 Weiss Hall, Department of Psychology, Temple University, 1701 N. 13th Street, Philadelphia, PA 19122 USA; 2 Department of Psychology, University of Illinois at Chicago, 1007 W. Harrison Street M/C 285, Chicago, IL 60607 USA; 3 Department of Computer Science, University of Illinois at Chicago, 851 S. Morgan Street M/C 152, Chicago, IL 60607 USA

**Keywords:** Embodiment, Spatial skills, Learning technology, Science learning, Geoscience

## Abstract

Although desktop simulations can be useful in representing scientific phenomena during inquiry activities, they do not allow students to embody or contextualize the spatial aspects of those phenomena. One learning technology that does attempt to combine embodiment and grounded experience to support learning in science is Embedded Phenomena. The objective of this research was to investigate the effectiveness of a classroom-based Embedded Phenomena activity for learning in geoscience, and to investigate whether individual differences in spatial skills had an impact on the effectiveness. The simulated scientific phenomenon was earthquakes, and 44 fifth grade (10-year old) students learned from a unit containing both content instruction and simulations. In the embedded condition, 15 earthquake events were simulated within the classroom space and students enacted the computation of epicenters with strings and their bodies. Students in the non-embedded condition received the same content instruction and did the same activities, but the epicenter computations were done with maps instead of with students’ bodies. Students in the embedded condition showed greater learning gains overall. Further, the Embedded Phenomena activity attenuated the effect of individual differences in spatial skills on learning in science such that low spatial individuals performed as well as high spatial individuals in the embedded condition.

## Significance

The pace of scientific research is accelerating and the average citizen is increasingly faced with having to understand and reason about matters of science in their everyday lives. Because it is imperative that the public be engaged in science issues that have an impact on them and their communities, it is therefore also important that the research community develop more effective methods of teaching science. More specifically, it is important that science education research focuses on methods for improving access and learning for all students including those who may find learning about science to be challenging. The findings of the current study are significant because they indicate that integrating technology, embodiment, and enactment in a science learning activity can be particularly beneficial for students who have weaker spatial skills. This finding is important because it suggests that changes in instruction can have an impact on student success and may allow students who may typically face challenges when learning about science to succeed. In a world where understanding matters of science is relevant to all our lives, it is important that all students have equal access to the opportunity to learn about science.

## Background

Spatial skills can predict learning in science, technology, engineering, and mathematics (STEM) particularly when understanding depends on the learner’s ability to visualize, manipulate, and animate spatial information (Hegarty, 2010; Uttal & Cohen, 2012). For example, developing mental representations of scientific phenomena may require spatial skills when entities or systems are not directly observable due to their spatial or temporal scale. In addition, constructing mental models of some scientific phenomena may involve representation of the configuration of multiple components, the spatial relations between components, or information about the movements or causal interactions among the components (Gentner & Stevens, 1983; Hegarty & Just, 1993; Mayer, 1989). In the case of plate tectonics, the student needs to create a mental model of a process that is too large to actually be observed. They must accurately represent the layers of the earth and the locations of tectonic plates, as well as information about how those plates move and interact with each other to cause phenomena such as earthquakes. Consistent with this analysis, prior work has demonstrated that spatial skills can predict learning in earth sciences and geology (Black, 2005; Jaeger, Taylor, & Wiley, 2016; Sanchez, 2012; Sanchez & Wiley, 2010).

Given that performance on tests of spatial skills often predicts achievement in STEM domains (Uttal & Cohen, 2012), it is important to understand the conditions that may allow students with weaker spatial skills to also succeed. Students who are low in spatial skills may struggle to create mental representations of spatial information on their own; therefore, conditions that support the representation of spatial information may be one route to improving student understanding. For example, some studies have shown that providing students with visualizations of scientific phenomena can be helpful. When benefits are seen specifically among low spatial students, it has been suggested that these external supports aid learning because these students are less able to create “runnable” mental models of phenomena on their own, and that the visualization provides a grounding or basis for mental model construction. For example, Sanchez and Wiley (2010) found that presenting relevant animations alongside an expository text about plate tectonics effectively eliminated performance differences between male and female students, even though the male students showed higher performance on tests of spatial skills. While providing visualizations to learners does not always help the low spatial students to learn more (Höffler, 2010), sometimes it fulfills a compensatory role such that low spatial students’ understanding benefits from the external support or grounding that is provided by visualizations. The present study explores whether two other types of grounding (embodying through action, and embedding in classroom space) may serve to improve learning.

One approach that has been shown to benefit learning in general, but may also reduce the need for spatial skills when learning science, is grounding student understanding via action or gesture (Lindgren & Johnson-Glenberg, 2013). Previous research has indicated that gesture is well suited to capture spatial information and is frequently produced when talking about space (Kita & Özyürek, 2003; Lavergne & Kimura, 1987). Further, because gesture is an action it may be a natural medium for aiding the creation of mental models that involve action (Goldin-Meadow & Beilock, 2010). From an embodied cognition perspective, it is suggested that students’ experiences with acting out specific events may allow them to ground their understanding within rich sensorimotor experiences (Kontra, Albert, & Beilock, 2012). For example, Kontra, Goldin-Meadow, and Beilock (2012) conducted a study in which college undergraduates learned about angular momentum either by manipulating a pair of bicycle wheels on an axle, or by receiving verbal descriptions while observing others manipulate the wheels. Although both the *observation* and *action* groups were matched on understanding at pre-test, students in the *action* group improved significantly at post-test, whereas students in the *observation* group did not. Further, action or gesture may help students to generate spatial mental models and more effectively maintain those models in memory (de Ruiter: Gesture and speech production, unpublished; Morsella & Krauss, 2004; Wesp, Hess, Keutmann, & Wheaton, 2001). de Ruiter had students verbally describe an array of shapes and lines either with the array visible or after it was removed from sight. Speakers produced more gestures when the array was no longer visible, which was taken to suggest that the gestures helped them to retrieve and maintain the mental image of the array.

Action or gesture may also help direct attention to and promote a focus on spatial information. Alibali, Spencer, Knox, and Kita (2011) showed that when students were allowed to gesture during a gear movement task they tended to use depictive strategies, modeling the movement of the gears with their gestures. When gesturing was not allowed, students generated verbal rule-based strategies. While these different strategies did not impact problem-solving success (both groups solved the same number of items correctly), gesturing biased students toward considering more spatial aspects of the problem. This suggests that the action information expressed in gesture may influence thought by adding spatial information to one’s mental representations. Other recent work has demonstrated that hand gestures can serve as an external support for maintaining spatial representations and that the frequency of gesture use is correlated with having low spatial skills (Chu & Kita, 2011).

A complementary approach to grounding learning in action is grounding learning within familiar or concrete experiences. Similar to the benefits of enactment, this approach would suggest that when a simulation takes place within a familiar space, such as the student’s classroom, that it may be easier to begin to represent spatial information than when a simulation is contrived to take place in a less familiar or more abstract other space. Simulations that are contextualized within a familiar space may be advantageous because they provide a strong and intuitive link between the elements of the real world and the elements of the hypothetical world (Fyfe, McNeil, Son, & Goldstone, 2014; Goldstone & Son, 2005; Kotovsky & Gentner, 1996). As suggested by the theory of concreteness fading, starting with a concrete context and then gradually removing context-specific elements can support learning and transfer. Specifically, more contextualized learning activities can provide a concrete context on which to ground understanding of concepts that might otherwise be more abstract and less readily comprehended, which should benefit learning on the whole, but may also be especially beneficial for low spatial students.

Taken together, instructional approaches that provide grounding for learning in two complementary ways – embodying through action, and embedding through classroom space – would seem to be a promising avenue for providing low spatial students with the support they need to learn complex scientific phenomena. The Embedded Phenomena framework is one such approach that attempts to combine embodiment and grounded experience to support learning in science (Moher, 2006). Two central components of the Embedded Phenomena framework are that technology-enhanced simulations are grounded by mapping them to the confines of the classroom itself and that simulations involve students as enactive agents. The framework has been used as the basis for a variety of hybrid simulation activities in domains spanning astronomy (HelioRoom; Thompson & Moher, 2006), population ecology (WallCology; Moher, Uphoff, Bhatt, López Silva, & Malcolm, 2008), and hydrology (AquaRoom; Novellis & Moher, 2011). While the Embedded Phenomena framework has previously been implemented in elementary classrooms, none of these investigations has specifically tested for effects of the simulation activities on domain-specific conceptual learning, or the relationship of learning with students’ spatial thinking skills. These were the goals of the current investigation.

**Fig. 1:**
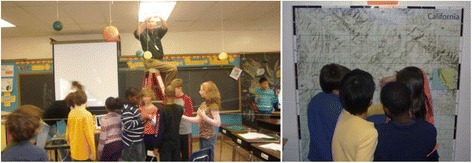
*Left*: epicenter location using students’ bodies and polystyrene balls in embedded class. *Right*: epicenter location using a map and stickers in the non-embedded class

In the current investigation the simulated scientific phenomenon was earthquakes, and students from two fifth grade classrooms learned from a unit called RoomQuake that contained both content instruction and simulations. In both embedded and non-embedded conditions, students collected earthquake data and contributed to shared aggregate representations of earthquake magnitudes and locations. In the embedded condition, the 15 earthquake events happened sporadically and unexpectedly over a 4-week period and all public data representations were persistent and present throughout. When an event occurred, students moved from their seats to go gather wave information from seismic reading stations distributed around the classroom space, and then enacted the computation of epicenters with strings and their bodies (see Fig. 1). Meanwhile, students in the non-embedded condition received the same content instruction and did the same activities, but the epicenter computations were done with maps instead of with students’ bodies. Further, the earthquake events were not embedded in classroom space, students completed the epicenter computations for all 15 events as one activity, and public data representations and traces were only present in class for those days.

The primary learning goals for the RoomQuake unit included developing an understanding of earth science concepts such as the layers of the earth, plate tectonics, and convection currents, as well as more specific concepts related to the characteristics of earthquakes, such as how they are distributed and how they are measured. Because spatial thinking skills have been shown to contribute to successful learning in STEM, and more specifically geosciences, an additional goal was to investigate the impact of different versions of RoomQuake on learning as a function of spatial thinking skills.

Because learning about earthquakes is both highly spatial and abstract (in that earthquakes involve processes too large to be actually observed) it was hypothesized that students in the embedded condition would show greater learning gains than those in the non-embedded condition. This hypothesis was motivated by the idea that embodiment and contextual grounding, as provided in the embedded condition, can be especially beneficial for developing a mental model of complex spatial information and would provide the support needed for abstraction and transfer more generally. Further, it was hypothesized that the potential effects of spatial skills on learning about plate tectonics would be reduced in the Embedded Phenomena condition. With Embedded Phenomena, the need to mentally visualize and manipulate representations of the phenomena should be reduced because the enacted activity provides a basis or grounding for the construction of mental representations. Students in the Embedded Phenomena classroom with weaker spatial skills should show equal learning outcomes to students high in spatial skills. However, in the non-embedded condition, it was expected that spatial skill would predict learning. Specifically, it was hypothesized that students with higher spatial skills would outperform students with lower spatial skills, consistent with prior work showing the effects of spatial skills on learning this content.

## Methods

### Participants

In a diverse Midwestern elementary school (67.7% white) in the USA, 44 fifth grade students (10-year olds) participated as intact classes across 2 consecutive years with the same teacher. There were 26 students (56% boys) in the Embedded Phenomena condition and 18 students (50% boys) in the non-embedded condition. No differences were seen between boys (*M* = 5.09, *SD* = 2.22) and girls in spatial skills (*M* = 4.65, *SD* = 2.08, *t* <1), which replicates previous findings for this age group (Voyer, Voyer, & Bryden, 1995). Further, the embedded and non-embedded classes were matched on spatial skills as well as on earth science concepts and earthquake measurement at pre-test, all *t*s <1.10.

### Manipulation and procedure

**Fig. 2:**
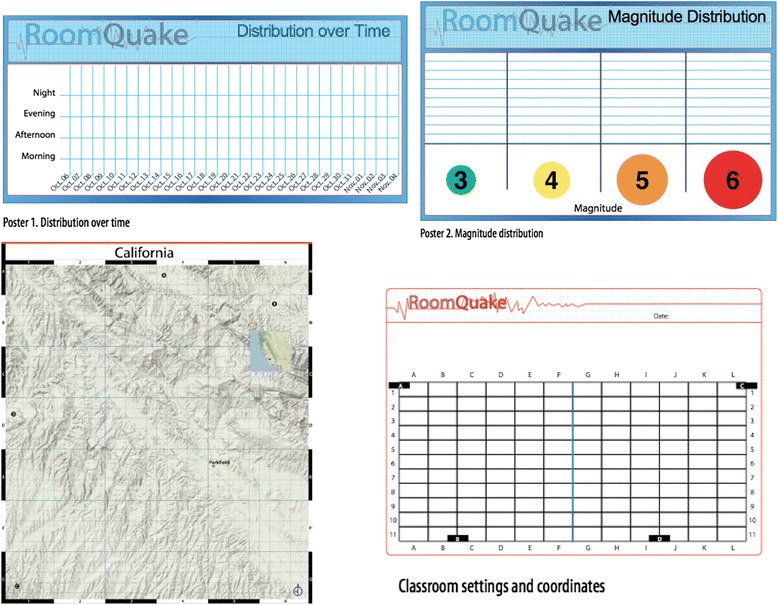
Examples of the multiple data representations in public displays that students used during the unit. Both classes used the same representation to record the date and time of earthquake events (*top left)* and event magnitude (*top right*). In the non-embedded class students recorded earthquake location on a map of California (*bottom left*) and in the embedded class they recorded RoomQuake locations on a map of the classroom (*bottom right*)

The two classes were followed over a period of 6 weeks as they participated in an earth science unit on the understanding of concepts about the earth’s layers and composition, the existence of tectonic plates, convection currents, plate boundaries, and the geological features that relate to the interactions of tectonic plates. Both classes received the same lessons on these topics during their regularly scheduled science classes. A breakdown of the lessons and differences across the two classes can be found in the Appendix. In addition, both classes completed a series of 15 simulated earthquake event activities in which they were required to compute and locate earthquake epicenters from seismograph data, and record this data in multiple representational formats. These activities were designed to support the acquisition of knowledge about earthquake measurement and skills in authentic seismological practice, including the determination of event distance and magnitude, and the use of trilateration to determine event epicenters. The representation of the data in multiple formats in persistent public classroom displays was intended to support the development of an understanding of the distributional characteristics of earthquakes across the dimensions of space, intensity, and time (see Fig. 2).

**Fig. 3:**
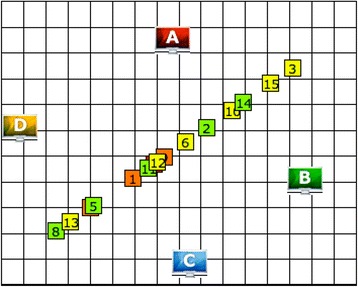
A schematic depiction of the embedded condition classroom layout. The *squares marked with letters* (*A*, *B*, *C*, *D*) represent the locations of the four iMac computer reading stations. The *squares marked with numbers* (*1* through to *15*) represent the approximate epicenter locations of the simulated earthquake events

The main manipulation between the two classes was in how the students engaged in the earthquake activities. Students in the Embedded Phenomena condition experienced the simulated earthquakes (“RoomQuakes”) as being located within the physical space of the classroom. These RoomQuakes occurred at random times throughout the school day over a period of 4 weeks (no RoomQuakes occurred during the first and last weeks of the unit). Specifically, five of the RoomQuakes occurred during the regularly scheduled science class, seven occurred during other subjects throughout the school day, and three occurred out of school hours. The RoomQuakes were created by placing four 24-inch iMac computers and speakers around the classroom (see Fig. 3). Each computer served as a seismograph reading station, which depicted a continuously running strip chart recorder of ground vibration. When a RoomQuake occurred, the seismographs traced out characteristic waveforms (seismograms) that corresponded to the expected vibration at their specific locations due to an event at a particular location in the classroom, and a rumbling sound was generated by the speakers in the iMacs. In the time between events, the seismographs displayed random visual noise and no sound. When the rumbling began, students ran to collect data from each seismograph.

In the embedded condition, when a RoomQuake occurred during a non-science class the students would get up from their seats and go to their stations to begin the process of measuring the waves and using trilateration to locate the epicenter just as they would have if it occurred during their science class. After completing the task, they would go back to their regularly scheduled subject. To track the occurrence of RoomQuakes outside school hours, students would check the activity of the seismographs first thing each morning. If a RoomQuake had occurred there would be a recorded seismogram available at each station that included the waveform that was created, the date, and time of the event so students could measure and record the event. Having RoomQuakes occur during non-science classes and on evenings and weekends was intended to reinforce the idea that earthquakes are not predictable and can happen at any time.

Students in the non-embedded condition also worked with the same earthquake data, but in their case it was presented as a series of 15 historical earthquakes that had occurred in 1999 in Southern California. For this condition, the iMac computers were placed in a row with each representing a different reading station that was suggested to be located in Southern California and identified on a map. Students were able to go forward and backward through an archive of “snapshots” of the seismograms taken at each location during each earthquake event. The 15 earthquake activities were completed as part of a single lesson spanning three science class sessions. Although the earthquakes in this class were presented as historical data from California, the data matched that of the embedded class. Specifically, both classes had the same patterns of magnitude (more small earthquakes than large), time (large earthquakes tended to be followed closely in time by several smaller ones), and location (occurred along a fault line).

The actual process for determining epicenters (trilateration) also differed between conditions. The embedded class used calibrated dry-lines that were anchored at each of the seismograph reading stations. To determine the distance each reading station was from the event epicenter, students had to measure the length of the primary wave. The length of this wave was then used to calculate the length of the dry-line. One quarter of an inch (6.35 mm) was equal to 1 pre-determined unit of dry-line (approximately 1 foot; 304.8 mm) so, if the wave measured 2 inches (50.8 mm) the students needed to measure out 8 units of dry-line. Once each group determined the appropriate length of dry-line for the event, the group would sweep out circles with their bodies, which reflected the possible loci of solutions from the individual stations until they found the place where all four dry-lines (and bodies) coincided. The place where students converged represented the epicenter of the event. The non-embedded class used calibrated dry-lines that were pinned to a large map of Southern California (see Fig. 1). Again, one quarter of an inch (6.35 mm) of length for the primary wave was equal to 1 pre-measured unit of dry-line; however, in this condition 1 unit of dry-line was approximately 1 inch (25.4 mm) in length. Despite the relative difference in scale across the two conditions (whole classroom trilateration versus wall map trilateration), the entire process of measuring, locating, and recording an earthquake event took approximately 12 to 15 minutes in both conditions. This suggests that the difference in scale did not have an impact on the difficultly of completing the trilateration activities.

**Fig. 4:**
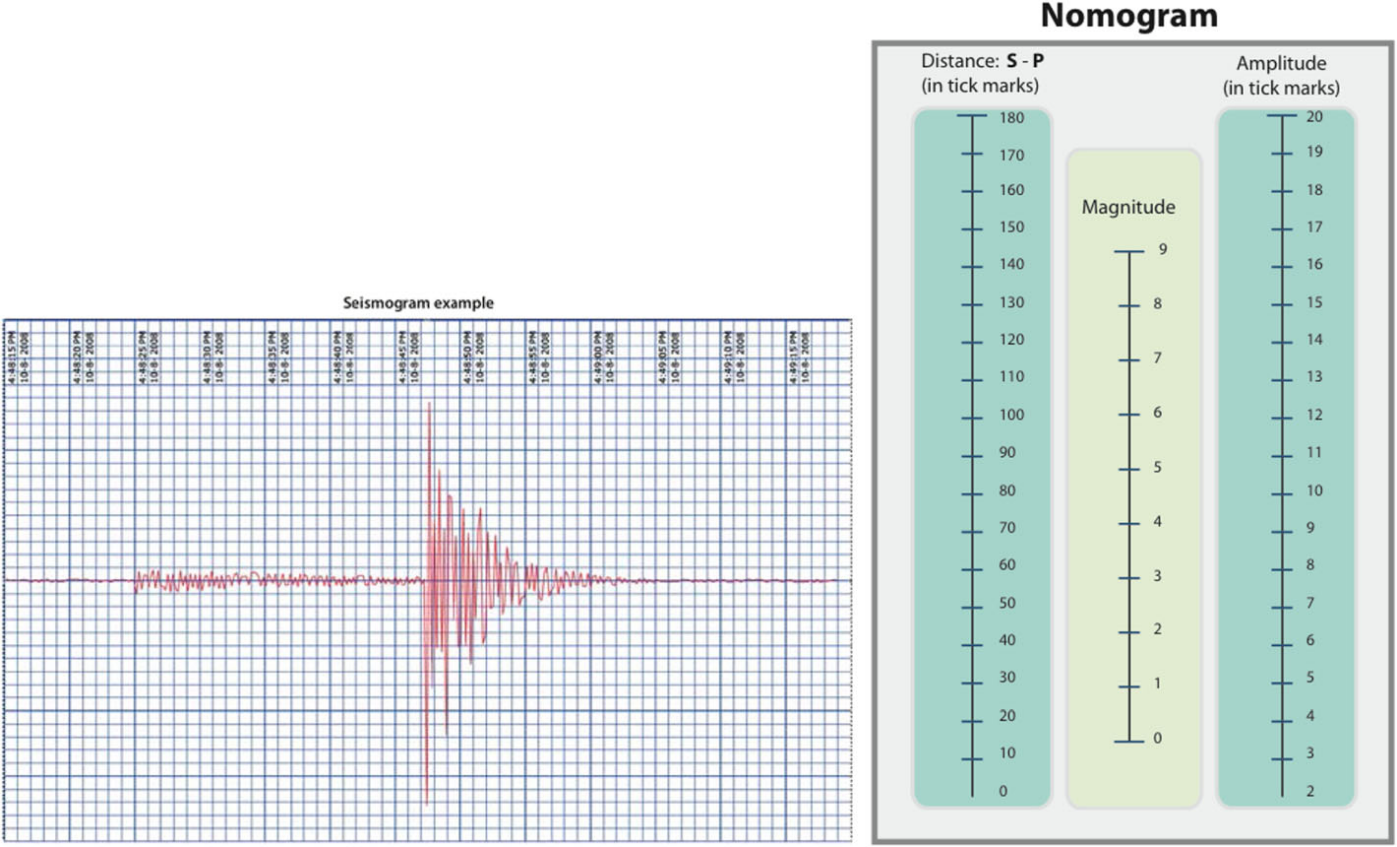
An example seismogram (*left*) where the first, shorter wave is the primary wave and the second, taller wave is the secondary wave. Students used the primary and secondary wave measurements to determine the magnitude using the nomogram (*right*)

The process for determining event magnitude was the same across both conditions. In order to determine event magnitude, students had to measure the amplitude (or height) of the secondary wave. Both the height of the secondary wave (amplitude) and the length of the primary wave (distance) were required to determine the magnitude with a nomogram (see Fig. 4). Students marked the distance on the left of the nomogram and the amplitude on the right of the nomogram, then using a straightedge would connect those two marks. The point at which the line crossed the center on the nomogram would give students the magnitude of the event.

Students in both conditions were responsible for recording event data in multiple formats. They entered it in their individual workbooks and on public displays in the classroom, which included the magnitude of each event, the location, and a timeline for when each event occurred. In the Embedded Phenomena class, the location of each event was marked by hanging a polystyrene ball from the ceiling where the class determined the epicenter of that event to be. Each ball was color-coded to reflect the magnitude of that event. Students in the embedded class also recorded this data on a classroom map in their workbooks. In the non-embedded class, students recorded epicenter locations on a large map of Southern California and used color-coded stickers to reflect the magnitude. They also recorded this data on a small map of Southern California in their workbooks. Both classes completed the same “big ideas” worksheets and engaged in whole-class discussions about the earthquake simulation activities; the discussions were intended to help them reflect on the lessons, map across representations, and connect data collection activities to conceptual understanding. For an overview showing the differences between the two conditions see the Appendix.

Because the teacher, and not the research team, carried out the activities it was important that the teacher be consistent across both conditions. Therefore, two intact classes across 2 consecutive years served as the sample for this study, and the same teacher implemented both conditions. The teacher had extensive experience with the unit and had been involved in running pilot versions of both conditions in their classroom for several years prior to this study. The teacher also worked collaboratively with the research team, including a seismologist, to develop the learning goals, activities, lessons, and assessments for the unit. This collaboration was essential because it ensured that the lessons were scientifically accurate according to the seismologist, but also age and curriculum appropriate according to the teacher.

### Measures

There were three primary learning goals for this unit. The first learning goal was related to developing an understanding of basic earth science concepts. This included understanding concepts about the earth’s layers and composition, the existence of tectonic plates, convection currents, plate boundaries, and the geological features that relate to the interactions of tectonic plates. The second learning goal was related to understanding the methods used in observing and measuring earthquakes as well as characteristics of earthquake data. For example, students were expected to develop an understanding of the distributional characteristics of earthquakes across the dimensions of space, intensity, and time and understand how information from wave information is used to determine these characteristics. The third learning goal was related to developing authentic skills in seismological practice. This included demonstrating skill in being able to compute and locate earthquake epicenters from seismograph data, determine event distance and magnitude, and use trilateration to determine event epicenters.

In order to examine the effectiveness of different versions of this unit for achieving the aforementioned learning goals, several sources of data were collected. A 20-item multiple-choice test was developed and given to both classes prior to beginning the unit and after the unit was completed. Ten of the items were related to understanding of the target earth science concepts and ten were related to understanding the methods used in observing and measuring earthquakes. The assessments were developed in a collaborative effort by the classroom teacher, based on his standard curriculum, an expert seismologist, and the rest of the research team. The pre-test and post-test items were designed using standard items from the content of the existing curriculum, including items found on standardized assessments from the National Assessment of Educational Progress (NAEP) and Illinois Standards Achievement Test (ISAT).

In addition, students’ seismological skills and their ability to work with multiple representations of data were assessed through hands-on skills assessments conducted in individual sessions with an experimenter. This post-unit assessment tested a student’s ability to locate the arrival of the primary and secondary waves, determine the distance between the arrivals of those waves, the amplitude of the earthquake, and the magnitude of the earthquake. In addition, students were asked to show the loci of potential epicenters using two different methods. First they had to find the epicenter by means of the method used in their class (either with strings in the classroom or strings on a map) and then they were asked to find an epicenter on a transfer task using three compasses on a piece of graph paper. There was a total of seven possible points. Both multiple-choice and skills assessments were piloted and revised to ensure their alignment with and coverage of the learning goals of the unit, and their appropriateness for the ability level of the students.

Prior to the beginning of the unit, students completed a 10-item paper-folding test (Ekstrom, French, Harman, & Derman, 1976). In this test, participants select which one of five possible patterns of holes will result after a piece of paper is folded and a hole is punched through it. Participants’ scores were computed as the number of correct responses. This test was chosen because it has commonly been used as a measure of spatial visualization skill, representing one’s ability to mentally transform or manipulate objects (Carroll, 1993). More specifically, it requires the manipulation of an internal representation as well as the transformation of three-dimensional elements. These task demands align with the demands of creating a mental representation of a fault line from continuously updating earthquake data; both incorporate three-dimensional information and both incorporate updating a mental representation through multiple transformation stages. Further, the paper-folding test has been demonstrated to be a strong predictor of performance or aptitude in STEM areas (Höffler & Leutner, 2007; Hsi, Linn, & Bell, 1997; Lord, 1987; Mayer & Sims; 1994; Siemonhowski & MacKnight, 1971) and, more specifically, in geosciences learning (Black, 2005; Jaeger et al., 2016; Sanchez, 2012; Sanchez & Wiley, 2010).

## Results

The current study examined whether embedding scientific phenomena within the space of the classroom would increase students’ knowledge of earth science concepts, earthquake measurement, and seismological skills beyond that of a non-embedded control condition. Furthermore, the study also investigated the role of spatial skills in learning in an Embedded Phenomenon setting. Repeated measures analysis of variance (ANOVA) was used to determine the effect of embedding a condition on various learning and skill outcomes. In order to analyze the effects of spatial skills on learning outcomes in the embedded condition and in the non-embedded condition, linear regressions were conducted and interactions were followed up with tests of simple slopes.

A repeated measures ANOVA on earth science concepts revealed an overall increase from pre-test to post-test, *F*(1, 42) = 11.86, *MSE* = 1.73, *p* < .01. Although there was no main effect for embedding condition, *F*(1, 42) = 1.90, *ns*, a significant interaction was observed, *F*(1, 42) = 11.86, *MSE* = 1.73 *p* < .01, indicating that while the conditions did not differ at pre-test, students in the embedded condition performed better at post-test than students in the non-embedded condition.

**Fig. 5:**
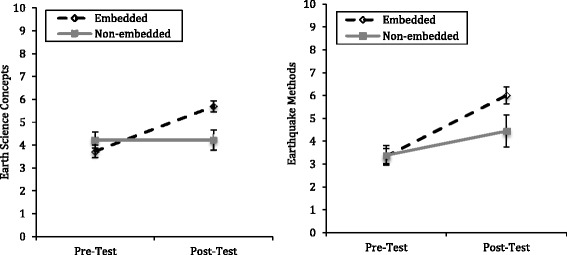
Relationship between earth science concepts performance at pre-test and earth science concepts performance at post-test as a function of embedding condition (*dashed line*, embedded; *solid line*, non-embedded) in the *left* panel. Relationship between earthquake methods performance at pre-test and earthquake methods performance at post-test as a function of embedding condition (*dashed line*, embedded; *solid line*, non-embedded) in the *right* panel

The same pattern of effects was found with a second repeated measures ANOVA on the earthquake methods items. A significant increase was seen from pre-test to post-test, *F*(1, 42) = 41.30, *MSE* = 1.77, *p* < .001, no main effect was seen for embedding condition, *F*(1, 42) = 1.77, *ns*, and there was again a significant interaction indicating that while there were no differences at pre-test on the earthquake methods items, students in the embedded condition performed better at post-test than students in the non-embedded condition, *F*(1, 42) = 7.69, *MSE* = 1.77, *p* < .01. The patterns of means for all learning measures are shown in Fig. 5 by condition.

**Fig. 6:**
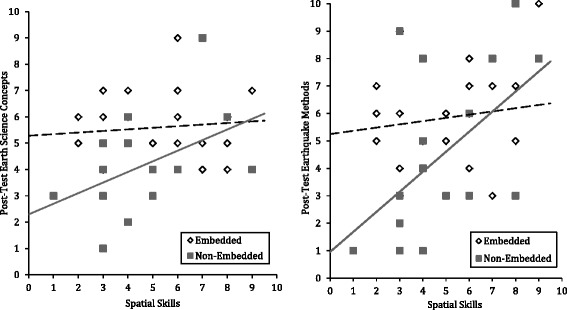
Relationship between spatial skills and performance on the earth science concepts items at post-test by embedding condition (*dashed line*, embedded; *solid line*, non-embedded) in the *left* panel. Relationship between spatial skills and performance on the earthquake methods items at post-test as a function of embedding condition (*dashed line*, embedded; *solid line*, non-embedded) in the *right* panel

Next, a series of linear regressions were used to analyze the effect of the Embedded Phenomena condition and spatial skills on learning outcomes. First, a linear regression examining the effects of spatial skills, condition, and their interaction on earth science concept learning was conducted. The model significantly predicted performance on the earth science concept items at post-test, *R* ^*2*^ = .29, *F*(3, 41) = 5.14, *MSE* = 2.10, *p* < .01. There was a main effect for embedding condition such that students in the Embedded Phenomenon condition learned more than students in the non-embedded condition, β = .91, *t* = 2.64, *p* < .02). There was also a main effect of spatial skills indicating that spatial skills predicted learning of earth science concepts (β = .52, *t* = 2.48, *p* < .02), and there was a marginal interaction (β = –.62, *t* = 1.63, *p* < .11 see Fig. 6, left panel). Based on *a priori* predictions that the embedded condition would be especially beneficial for low spatial students, the marginal interaction was followed-up with a test of simple slopes. The tests of simple slopes revealed that students with higher spatial skills showed better performance in the non-embedded condition (β = .34, *t* = 2.48, *p* < .02), but not in the embedded condition (β = .06, *t* = .44, *ns*).

A second linear regression was conducted to examine the effects of spatial skills, condition, and their interaction on earthquake methods learning. The regression model including spatial skills, condition, and their interaction significantly predicted performance on the earthquake methods items at post-test, *R* ^*2*^ = .26, *F*(3, 41) = 4.33, *MSE* = 4.90, *p* < .01 (Fig. 6, right panel). Students in the embedded condition did better on the methods items than students in the non-embedded condition, β = .87, *t* = 2.48, *p* < .02. There was also a main effect of spatial skills indicating that spatial skills predicted learning of earthquake methods (β = .63, *t* = 2.94, *p* < .01), and a marginal interaction (β = –.77, *t* = 1.87, *p* < .07). Again, to address *a priori* predictions that embedding would be especially beneficial for low spatial students, the marginal interaction was followed-up with a test of simple slopes. The tests of simple slopes again revealed significant effects of spatial ability on performance in the embedded condition (β = .41, *t* = 2.94, *p* < .01), but not in the Embedded Phenomena condition (β = .08, *t* = .56, *ns*).

**Fig. 7:**
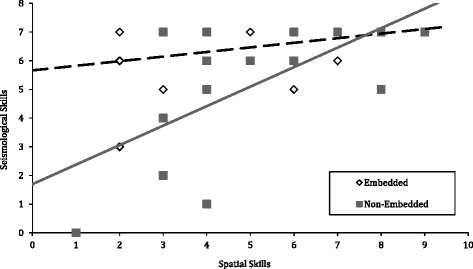
Relationship between spatial skills and performance on the seismological skills items at post-test as a function of embedding condition (*dashed line*, embedded; *solid line*, non-embedded)

Finally, a third linear regression was conducted including spatial skills, embeddedness condition, and their interaction and it significantly predicted performance on the seismological skills assessment, *R* ^*2*^ = .46, *F*(3, 41) = 10.84, *MSE* = 1.94, *p* < .001 (Fig. 7). Students in the embedded condition performed better on the skills assessment (*M* = 6.46, *SD* = .99) than students in the non-embedded condition (*M* = 4.94, *SD* = 2.28), β = .39, *t* = 3.30, *p* < .01. Spatial skills also predicted learning of seismological skills (β = .80, *t* = 4.35, *p* < .001), and there was a significant interaction (β = –.46, *t* = 2.53, *p* < .02). Tests of simple slopes again revealed significant effects of spatial ability on performance in the non-embedded condition (β = .52, *t* = 4.35, *p* < .001), but not in the Embedded Phenomena condition (β = .14, *t* = 1.20, *ns*).

## Discussion

This experiment followed two classes of students across a 6-week unit in earth science to investigate the effects of the Embedded Phenomena approach. Analyses revealed that at pre-test, students in the embedded and non-embedded conditions were matched on their knowledge about the content of the unit. In addition, there was an overall significant increase on these items from pre-test to post-test suggesting that in both conditions students did learn the content. However, the gains in the Embedded Phenomena condition were significantly larger than the gains seen in the non-embedded condition.

Furthermore, this embedded enactive simulation of earthquake phenomena particularly improved the learning of students with weaker spatial skills. Results from this study demonstrated that spatial skill did constrain learning in the non-embedded conditions, but that the dependence on spatial skills can be reduced when an activity is embedded and enacted within classroom space. Specifically, in the Embedded Phenomena condition, students with low spatial skills were performing at a level equal to students with high spatial skills. This pattern of results suggests that grounding an activity in an embodied experience may lessen the demands of mentally representing the phenomena, which may be critical for supporting improved understanding of many scientific topics. Future work should examine whether certain items drove the improvement seen in the embedded condition and, in particular, whether test items that required mentally representing spatial attributes of the phenomena showed the strongest gains.

Two salient features that were intentionally varied between the embedded and non-embedded versions of the unit were the extent to which the activity was embedded and persistent within classroom space, and the extent to which it required students to enact the epicenter computations with their bodies. However, there was also another salient feature that differed between the two activity structures: whether the epicenter activities were done in a massed fashion (one after the other within a couple of class periods in the non-embedded condition) or distributed over time (in the embedded condition). While embedding the activity in classroom space and supporting it with enactment were both intended to support spatial understanding of earthquake phenomena, the decision to distribute earthquake events unexpectedly over time was intended to help students to understand the serendipitous, slow, and patient nature of real science. Because these two different classes of feature variations were not manipulated independently in the present study, this design might be conceived as representing only two conditions of a larger 2 × 2 design that would have fully dissociated the spatial embedding/embodying features of the activity from the temporal distribution of the activities. Given the limitations of the present design, it cannot be ruled out that the advantages of the embedded condition might really be due to the distributed nature of the epicenter activities. However, although it is possible that there was a benefit to spacing or distributing the activities over time on learning (Carpenter, Cepeda, Rohrer, Kang, & Pashler, 2012), it is not clear what theory would predict that manipulating this feature should facilitate the learning of individuals with weaker spatial skills. On the other hand, the interaction that was observed is predicted well by the assumption that the embedding and embodying features were facilitating learning by helping students to ground their learning experiences.

Similarly, it could be argued that the two activity structures also differed in the extent to which they were engaging or motivating for the students. From this perspective, the greater overall learning gains seen in the embedded condition could simply be due to the fact that students were more engaged and motivated to learn. Student responses from post-unit interviews were explored in an attempt to identify any differences in student engagement or enjoyment across conditions. Overall, students from both conditions reported liking or preferring whatever condition they had experienced and that the overall length of the unit was just right. When asked about how this unit compared to other science units they had done, almost all of the students in both conditions reported that this was more fun and helped them to learn more than traditional methods such as reading textbooks or watching videos. The two points where students in the embedded and non-embedded conditions seemed to differ was in how authentic they felt their experience to be. Students in the embedded classroom felt that their experience was similar to what seismologists do in real life: such that they experience earthquakes and try to locate them. On the other hand, students in the non-embedded condition did not feel that their experience was similar to what seismologists do in real life because they did not get to experience earthquakes. In addition, students in the non-embedded condition tended to describe their task as being one of doing research on a computer; no students reported this in the embedded condition. While it is possible that increased engagement or motivation could have had an impact on student learning overall (Hampden-Thompson & Bennett, 2013), and there is some evidence from the interviews that students in the embedded condition thought their experience was more authentic, it is again not clear that this would specifically facilitate the learning of individuals with weaker spatial skills.

One factor that makes this study unusual and interesting, but also inherently limited, is that it involved implementing an immersive technology framework within a real elementary school classroom context. Some of the limitations associated with being able to conduct this unit in authentic classrooms were that only a single experienced teacher was responsible for conducting the units and, although the teacher delivered all the content, the technology was heavily supported and controlled by the research team. This poses a potential limitation for broader dissemination because naïve teachers would require training to use it, and they might also need experience to use it as effectively as the teacher in the current study. However, the results were well aligned with theories of embodied cognition and previous research indicating that low spatial students can perform as well as high spatial students when given the appropriate supports (Sanchez & Wiley, 2010, 2014; Stieff, Dixon, Ryu, Kumi, & Hegarty, 2014). These results also align well with results from Jaeger et al. (2016) which showed that spatial skills are generally related to improved learning about another geoscience phenomenon (El Niño), but that providing an interleaved analogy can help support learning for low spatial students. Like the Embedded Phenomena simulation employed in the current study, an interleaved analogy can provide low spatial students with a familiar context on which to map a spatial mental model of a phenomenon, and this in turn reduces the need to rely on spatial skills for creating a mental model on their own. Another limitation worth noting is that the teacher was highly experienced with the unit and this experience may have been important for overall learning. However, the experience level of the teacher cannot account for the pattern of results showing that low spatial students learned better from the embedded condition.

More recent research using the Embedded Phenomena framework has been conducted with the goal of further exploring shared data displays for supporting spatial representations, as well as implementing new knowledge-construction activities, and making it easier to involve new teachers and train them to use the technology themselves (Moher et al., 2015). Specifically, newer iterations of RoomQuake focused on helping students to develop an understanding of the capabilities and limitations of seismographs, in particular, their dependence on the generation of multiple waves during an earthquake. This more recent iteration of RoomQuake introduces a knowledge-building progression in which a series of challenges is presented to students where they must determine the characteristics of earthquakes using a variety of tools including seismographs, tape measures, and stop watches. One major limitation of the version of RoomQuake used in the current study was that it required extensive teacher experience as well as support for the technology. In this newer iteration of RoomQuake, the classroom still serves as the experimental space, however, the teacher controls the simulation technology. Further, rather than students working in teams to report consensus readings from specific seismographs, the new version uses mobile devices where each student reports readings from multiple seismographs. As the readings are reported, they are depicted in an aggregate public display representing a map of the room showing the distance estimates from each seismograph, reinforcing the “intersection of circles” framing that is difficult to achieve at classroom scale because of the occluding walls.

In addition, the Embedded Phenomena framework has been extended to other science domains including ecology and engineering. In a recent application called Hunger Games, students are situated as rabbits foraging among a variety of food patches within the physical space of the classroom (Gnoli et al., 2014). The learning goals of this unit include the development of student understandings of foraging behaviors as a function of food resource depletion, competition, and predation. In another application called AquaRoom, students are situated as being in a town beneath which runs a network of aquifers. Children are challenged to recommend locations for new chemical plants with the goal being to minimize the impact of potential pollutants on the underground water supply.

## Conclusions

Previous work with Embedded Phenomena simulations has shown that they can support development of domain understandings and authentic scientific practice, facilitate positive attitudes towards science and conducting experiments, and increase student agency in finding things out through experimentation rather than from a teacher (Malcolm, Moher, Bhatt, Uphoff, & López Silva, 2008; Moher, 2008; Moher et al., 2008; Novellis & Moher, 2011). The current research extends prior findings by showing that Embedded Phenomena activities may differentially improve learning and attenuate the effects of individual differences in spatial skills on learning in science, relative to non-embedded activities. As Gibson, 1979, (pg. 223) stated, “We must perceive in order to move, but we must also move in order to perceive.” The Embedded Phenomena framework may offer unique benefits for engaging students in authentic practices, improving learning outcomes, and making science learning accessible to students with a wide variety of skill sets.

## Appendix

**Table 1:** Differences Across Embedded and Non-embedded Classes

	Embedded class	Non-embedded class
Technology	• 4 computers • Distributed around the room • Seismometers were always moving • Displays on all day • Displays on entire unit • Earthquakes happened at random times • Trilaterated using classroom space	• 4 computers • All in a row • Seismometers showed still pictures • Displays on only in science • Displays on only 2 weeks • Earthquakes happened in the past • Trilaterated with maps and strings
Time and class periods	• Approximately 16 class periods • Science 4 days a week • Science periods 40 minutes • Approximately 700 minutes of RoomQuake (RQ)	• 18 class periods • Science 3 days a week • Science periods 1 hour • Approximately 1000 minutes of RQ
Background story	• Quakes are happening in the school • Happening in real time • Seismometers are sensing vibrations all day • Students locate 15 quakes	• Quakes are happening in California • Happened years ago • Seismometers only show still pictures • Students locate 15 quakes
Lesson 1 – Introduction to RQ	• What I know about earthquakes • Discussion about what students know and are wondering about earthquakes	• Same as embedded
Lesson 2 –Measuring, recording, and trilaterating	• Lesson about trilateration, waves, and nomogram • New vocabulary introduced • Class discussion about big ideas from today	• Same as embedded
Lesson 3 – Finding epicenters	• Revisit lesson on trilateration, waves, and nomograms • Use sample seismograms to practice measuring waves • Use strings to demonstrate trilateration in the classroom • Class discussion about big ideas from today • Students should now be ready for a quake to happen at any time until the end of the unit • Quakes happen at random times throughout the rest of the lessons • Students enter data in field guides and on public displays • Class discussions after each RoomQuake is plotted to discuss any patterns that students may be seeing in the charts and maps	• Same as embedded • Same as embedded • Use overhead transparency of California to demonstrate trilateration on map • Students go to large wall maps and seismograms • They plot their first earthquake on the map and record the data in their field guides • The students will continue locating earthquakes until all have been plotted (this took a total of 5 science class periods) • Class discussions at the end of every science period to discuss any patterns that students may be seeing • After final quake is plotted have the entire class look at all the charts and maps that were created across the epicenter location activities and discuss what happened
Lesson 4 – Earthquake research	• Break class into 7 groups: tectonic plates/earthquake location, earth layers, measuring/locating, historic quakes, quake preparation/safety, faults/geographic features, seismic waves • Have students research their topic with provided library books • Record research notes in field guide • Each group creates a presentation to share with the class • Students take notes on other groups’ presentations	• Same as embedded
Lesson 5 – Earth’s layers	• Students draw a picture of earth’s layers only using prior knowledge • Teacher shows overhead of real layers • Lecture on layers and what they are made of • Students split into pairs and make clay models of earth’s layers	• Same as embedded
Lesson 6 – Plate tectonics	• Students write down their ideas about how the continents moved and about Pangea • Class discussion about continental drift versus plate tectonics • Teacher explains that plate tectonics is correct and explains convection currents • Make Pangea flip books • Return to big ideas and discuss questions students still may have	• Same as embedded
Lesson 7 – Tectonic boundaries	• Teacher explains the different types of plate boundaries • Teacher shows animations of each type of boundary from the usgs.org site • Snicker bar demonstration of boundaries • Students label the plate boundary pictures in their field guides and the geographic formations associated with each type • Return to big ideas, journal about today’s activities	• Same as embedded
Lesson 8 – Seismic waves	• Teacher shows overhead of “waves” field guide page • Class talks about the properties of the different types of waves • Speed and range of waves is discussed (5.5 magnitude quake can be felt all over the world) • Slinky demonstration to show differences between P and S waves • Class discusses historic earthquakes – teacher explains there is a 100% chance of an earthquake everyday • Big ideas, students’ journal about today’s lesson	• Same as embedded
Lesson 9 – Wrap up and closing	• Review of major topics from unit • Students fill out post-anticipation guide	• Same as embedded
